# Identification of a novel *HEXB* Mutation in an Iranian Family with suspected patient to GM2‐gangliosidoses

**DOI:** 10.1002/ccr3.3103

**Published:** 2020-08-11

**Authors:** Fatemeh Mansouri‐Movahed, Fatemeh Akhoundi, Parvaneh Nikpour, Masoud Garshasbi, Modjtaba Emadi‐Baygi

**Affiliations:** ^1^ Department of Genetics Faculty of Basic Sciences Shahrekord University Shahrekord Iran; ^2^ Department of Genetics and Molecular Biology Faculty of Medicine Isfahan University of Medical Sciences Isfahan Iran; ^3^ Child Growth and Development Research Center Research Institute for Primordial Prevention of Non‐Communicable Disease Isfahan University of Medical Sciences Isfahan Iran; ^4^ Department of Medical Genetics Faculty of Medical Sciences Tarbiat Modares University Tehran Iran; ^5^ Research Institute of Biotechnology Shahrekord University Shahrekord Iran

**Keywords:** GM2‐gangliosidoses, *HEXA*, *HEXB*, sandhoff, β‐hexosaminidase

## Abstract

Sandhoff disease is one of the GM2‐gangliosidoses which is caused by a mutation in the *HEXB* preventing the breakdown of GM2‐ganglioside. We report a novel *HEXB* variant in a family with a history of a dead girl with Sandhoff disease which was not found in controls.

## Graphical abstract

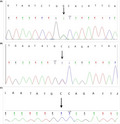

## 1 INTRODUCTION

The GM2‐gangliosidoses are autosomal recessive metabolic diseases caused by a reduced ability to metabolize the GM2‐ganglioside and result in ganglioside accumulation especially in the central nervous system.^1^, ^2^ The disorders show a spectrum of clinical severity. Generally, the earlier the age of onset of clinical symptoms is associated with the more severe form of the disease.^3^ The GM2‐gangliosidoses are caused by mutations in the genes encoding the subunit α (*HEXA* gene) or subunit β (*HEXB* gene) of heterodimeric b‐hexosaminidase A (*HEX A*), or its cofactor (the GM2 activator protein).^4^, ^5^ The lysosomal hydrolase, beta‐hexosaminidase, has two isoenzymes: hexosaminidase A and hexosaminidase B. Hexosaminidase A is composed of α and ß subunits, whereas hexosaminidase B is only composed of ß subunits.^6^, ^7^ Sandhoff disease^8^ (OMIMNo. 268800) caused by mutations of *HEXB* gene that contains 14 exons and spans about 40 kb of DNA on chromosome 5q13^9^ in which there is a deficiency of both lysosomal hydrolase β‐hexosaminidase A and β‐hexosaminidase B. This deficiency causes accumulation of GM2‐ganglioside especially in the neuronal cells.^4^, ^10^ In regard to the age at the onset of symptoms, three types of Sandhoff disease are distinguishable: infantile (classical Sandhoff disease), juvenile, and adult groups of patients.^2^, ^11^, ^12^ Sandhoff disease has several symptoms which may include motor weakness, hyperacusis, blindness, mental deterioration, cherry‐red spot in the macula, seizures, and optic atrophy.^13^ Tay‐Sachs disease (TSD) is a fatal neurodegenerative disorder manifesting the same ophthalmic symptoms found in Sandhoff disease, such as optic atrophy and a cherry‐red spot in the macula.^14^, ^15^ It is caused by mutations within the *HEXA* gene on chromosome 15q 23‐24.^1^ The aim of this study was to investigate and identify the plausible disease‐causing mutation in an Iranian family with the dead suspected infant having GM2‐gangliosidoses.

## 2 METHODS

### 2.1 Subjects

The family of this study had a history of an affected girl based on the clinical documents and family history was strongly suspected of Sandhoff or Tay‐Sachs disease. The parents were healthy and first cousin. They had 3 children: two normal boys and only a girl who was born via normal delivery without any pre/postnatal insults with good Apgar score and normal birth weight and head circumference.

She was completely normal until the age of 9 months (based on her parents’ claims), but thereafter they noticed she has a problem as she lost her motor skills such as turning over, sitting, and crawling. Later, she developed exaggerated startle reactions to loud noises and experienced seizures. At the age of 2 years, observing cherry‐red spots by an eye specialist in her confirms the diagnosis of GM2‐gangliosidoses. Despite taking different antiepileptic drugs, her seizure never gets controlled completely. After several times of hospitalization due to severe infections and fever, she died at the age of 3 years due to respiratory problems.

In this case‐control study, we had two groups including parents of a case with suspected GM2‐gangliosidoses and 100 controls from population‐matched Iranian subjects who had no disease in their family. After informed consent has been obtained from all participants, blood samples were collected. The experiments were approved by the Ethics Committee of Shahrekord University.

### 2.2 DNA genotyping

Genomic DNA from peripheral blood of the parent and 100 control samples was extracted using the Diatome kit (Isogen Laboratory Russia) according to the vendor's recommended protocol. For all exons of the *HEXA* and *HEXB* gene, oligonucleotide primers were designed using primer3.0 software (Tables 1 and 2); the accuracy and efficiency of the designed primers were verified with the BLAST server (Basic Local Alignment Search Tool) (https://blast.ncbi.nlm.nih.gov/Blast.cgi).

**Table 1:** Primers for amplification of *HEXB* exons

Exon	Primer sequence (5′→3′)	Amplicon size (bp)
1.	Forward: 5′GTCTGGGCTCTGCTCCTTTAC 3′	698
	Reverse: 5′CAAGAGCGTGGGTTCAGTC3′	
2	Forward: 5′GGCAGCATGGATTTGAGGAG3′	297
	Reverse: 5′CAGCGAGCACCTGGGATATA 3′	
3	Forward: 5′AATAGGTCATGTGCTTGGGAG3′	289
	Reverse: 5′GGATCTAAGGCGGCAAAGTTTT3′	
4,5	Forward: 5′ATGGGTACAATGAAGTAAAGCAC3′	766
	Reverse: 5′TCCCCTGTTCCAAACTACACA3′	
6	Forward: 5′GAGCCAAGATCATGCCACTG3′	798
	Reverse: 5′AACTAGGGGACACTGAGGGT3′	
7	Forward: 5′GCTGTCAAATATCAAATGCAAGC3′	295
	Reverse: 5′GGGTGACAGAACAAGACTCC3′	
8	Forward: 5′CAAAGAGGCAAAGAGACAGG 3′	537
	Reverse: 5′GTAGAGATGTGGTTTCAC3′	
9	Forward: 5′GGTGGTAAGGTAAAGAAAGCCA3′	412
	Reverse: 5′GGCAAAGGATGTAGAGAAAATGT 3′	
10	Forward: 5′GGCACCTCTCAAAATGCAAGA3′	818
	Reverse: 5′ACCCACAACACTTGGCCAA 3′	
12,13	Forward: 5′TGCCTCTGTGTATAAGCTTTGA3′	585
	Reverse: 5′TGGAGTTCTAAGTTACACCAACA3′	
14	Forward: 5′GCTGCAGGATGGTCGAGTAA3′	599
	Reverse: 5′GATGCCAGGCCTCTAAATGT3′	

**Table 2:** Primers for amplification of *HEXA* exons

Exon	Primer sequence (5′→3′)	Amplicon size (bp)
1	Forward: 5′TGGCCGGTTATTTACTGCTCT3′	600
	Forward: 5′CATAGGGCGTCTCTGGAGC3′	
2	Forward: 5′CTTTCCTTTACCACAGGCGT3′	300
	Forward: 5′CTCCAGGCCGAGCATCAG3′	
3	Forward: 5′CAAAGGCATTGGGAGCATCG3′	526
	Forward: 5′TCCACCAACACCAACCTTCC3′	
4	Forward: 5′AGTGGCTTCCTAATATCCCCT3′	384
	Forward: 5′AATCTACCAAACCAGTTTTCCAC3′	
5	Forward: 5′AAGAATCCTGGGAGAGTTGTC3′	236
	Forward: 5′CTCTTAAGTGTGAAGAAGGCCT3′	
6	Forward: 5′CTGAGAGCTCGCCCAACAT3′	244
	Forward: 5GCCACAGCCAGATTCAGACA3′	
7	Forward: 5′AGTCTTGTGGGCATTTTGAGT3′	299
	Forward: 5′TATGCCACTTCCATGAGCCA3′	
8	Forward: 5′GTGAGTGCCTAAGTGTGCCT3′	591
	Forward: 5′AGACAATTCTGTGCCCAGGG3′	
9,10	Forward: 5′CAGGTGACTAATCCCCAGGC3′	560
	Forward: 5′ACTGCTGGTGGCTTCTTCTC3′	
11,12	Forward: 5′GGAATCTCCTCAGCTTTGTGT3′	598
	Forward: 5′TGGGATTGGGTCTCTAAGGGA3′	
13	Forward: 5′TTGCTCAAGACCCAGCACAA3′	531
	Forward: 5′CCCACAGCTTGCTTACCTCA3′	
14	Forward: 5′TTCATGGACAGGACAGCACC3′	515
	Forward: 5′AATACTTTGCCCCACCCCAG3′	

Genotyping of all the exons was carried out using polymerase chain reaction (PCR) and direct sequencing methods. The reactions were performed in 25 µL volume containing 2 µL genomic DNA, 2.5 µL (10×) PCR buffer, 0.75 µL MgCL_2_ (50 mmol/L), 0.5 µL of four dNTPs (40 mmol/L), 0.5 µL of each primers (10 µmol/L) (Tables 1 and 2), and 0.25 µL Taq DNA polymerase (5 U/µL) (KBC, IRAN). Amplification was achieved by incubation in a BIOER DNA Thermal Cycler (TC‐XP‐G) for an initial denaturation of 5 minutes at 95°C, succeeded by 35 cycles of denaturation at 95°C for 40 seconds, annealing at 56°C for 40 seconds, and extension at 72°C for 30 seconds followed by final extension of 10 minutes at 72°C. Before sequencing analysis, PCR amplicons were assayed for size and purity by separation on 1% agarose gel, stained by DNA green viewer (Afratoos, Iran).

### 2.3 Bioinformatics analyses

To predict the possible effect(s) of the plausible mutation on protein function, we used online server SIFT (Sorting Intolerant From Tolerant) (http://sift.bii.a‐star.edu.sg/index.html),^16^ PROVEAN (Protein Variation Effect Analyzer) (http://provean.jcvi.org),^17^ PolyPhen 2.0 (http://genetics.bwh.harvard.edu/pph2),^18^ I‐Mutant 3.0 (http://gpcr2.biocomp.unibo.it/cgi/predictors/I‐Mutant3.0/IMutant3.0.cgi),^19^ and PHD‐SNP (http://gpcr.biocomp.unibo.it/cgi/predictors/PhD‐SNP/PhD‐SNP.cgi)^20^ as stated in Table 3. The results obtained from the different algorithms were clustered to increase the accuracy of the predictions.

**Table 3:** In silico approaches available as online tools

Server	Feature	Reference
SIFT	Sequence homology‐based tool detect damaging single amino acid substitutions. The amino acid substitution is predicted damaging when the score is ≤0.05 and tolerated if the score is >0.05	Magesh & Doss (2014); Ng & Henikoff^16^
PolyPhen 2.0	Sequence and structure‐based method that predicts the influence of amino acid substitution on the structure and function of proteins. The output levels of probably damaging and possibly damaging were classified as deleterious (≤0.5) and the benign level being classified as tolerated (≥0.5).	Adzhubei et al (^21^); Ramensky et al^18^
I‐Mutant 3.0	For the automatic prediction of protein stability changes caused by single amino acid substitution. output result: DDG < −0.5: Large Decrease of Stability DDG > 0.5: Large Increase of Stability −0.5 ≤ DDG ≤ 0.5: Neutral Stability	Capriotti et al^19^
PROVEAN	Estimates whether a protein variant affects protein function. A protein variant is predicted to be neutral or deleterious (default is −2.5)	Choi et al^17^; Manickam, Ravanan, Singh, & Talwar (^22^)
PhD‐SNP	SVM based on evolutionary information predictor of human deleterious single‐nucleotide polymorphisms	Capriotti et al^20^; Magesh & Doss (^23^)
HOPE Project	An online web server for studying the structural features of native protein and the variant models.	Venselaar, te Beek, Kuipers, Hekkelman, & Vriend (^24^)

## 3 RESULTS

**Figure 1:**
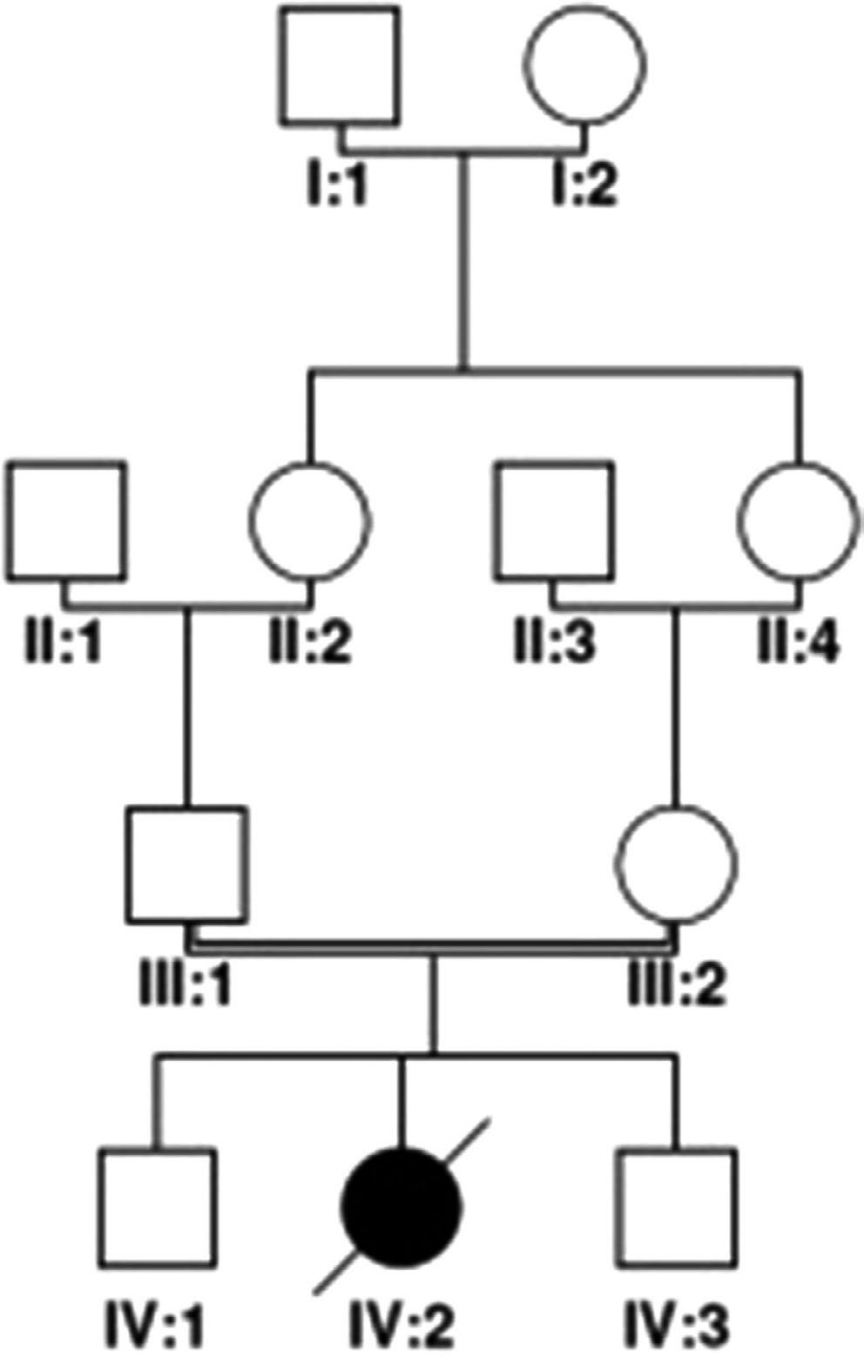
Family pedigree with GM2 gangliosidosis; drawing with Cyrillic software

The patient's family pedigree is shown in Figure 1. The control group included an equal number of men and women. First of all, since the symptoms exhibited by the dead girl were more likely to be signs of sandhoff disease, therefore, we started from *HEXB* gene which has 14 exons. Second, it was better to sequence firstly the exons which were more likely affected by mutation (s) and impact protein function that in turn lead to a severe form of the disease. Third, by considering the *HEXB* gene mutations in the GM2‐gangliosidoses database (http://data.mch.mcgill.ca/gm2‐gangliosidoses), we found that among the 14 exons of the gene, 4 exons including 7, 11, 13, and 14 have more mutations to take into account for further analysis. Fourth, the literature review has also revealed that among these 4 exons, mutations in exon 7 is more related to the acute form of GM2 gangliosidosis which is a devastating disease.^5^ Fifth, investigation in the three‐dimensional structure of beta‐hexosaminidase β‐subunit (*HEXB*) showed that exon 7 has less distance with the enzyme catalytic site and actually it is located in the vicinity of the enzyme active site.^25^, ^26^ Considering all the above reasons (first to fifth), we decided to begin our analysis from exon 7.

**Figure 2:**
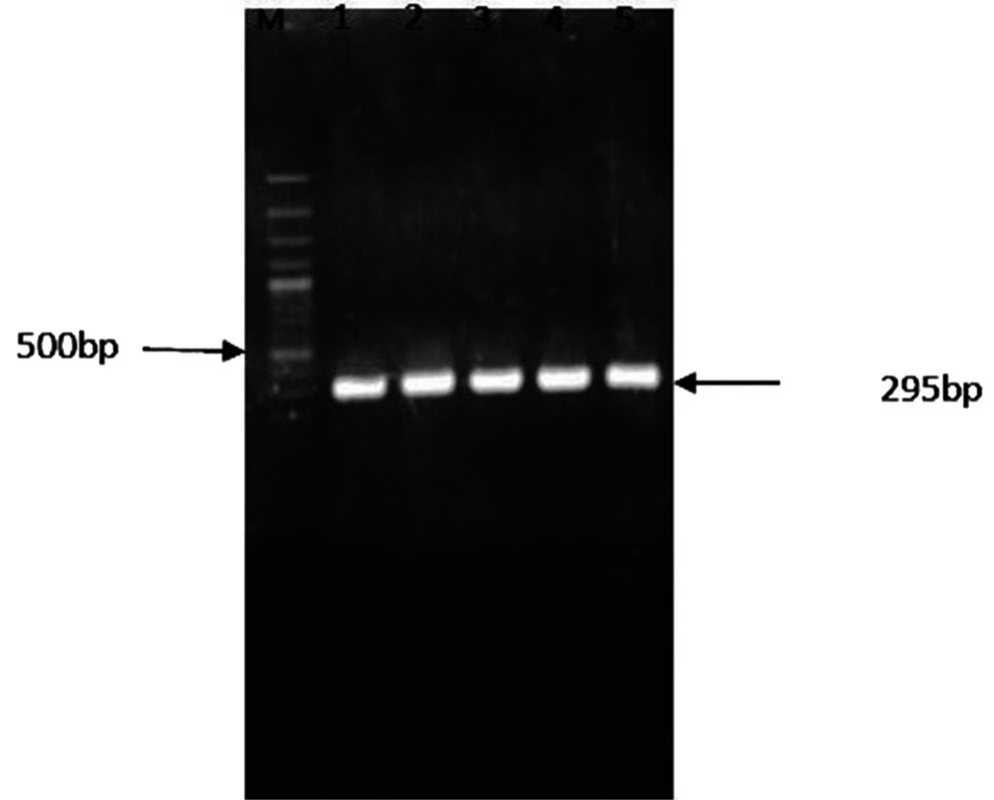
Electrophoresis of the 295 bp PCR products of exon 7 of the *HEXB* gene on an agarose gel. Lanes 1 and 2: the parents' of the dead infant (CT); Lanes 3, 4, and 5 are controls (CC). GeneRuler 100 bp DNA ladder from Fermentas (Catalog number SM0321) was used as the DNA ladder

**Figure 3:**
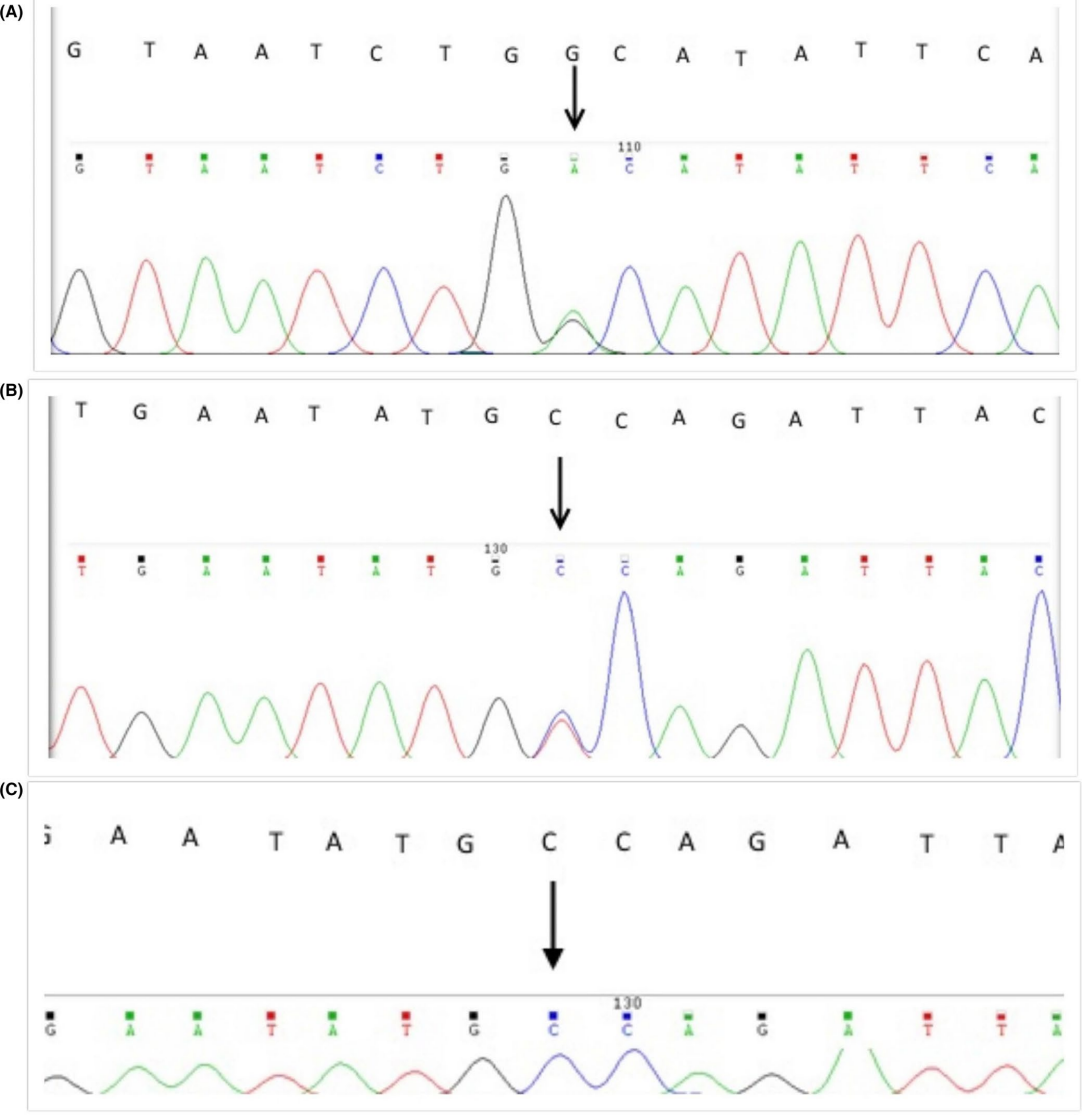
Results of the sequencing of exon 7 *HEXB* gene. a, b in the carriers(CT). Failure to match two sequences is due to the fact that one of the sequences is from the coding strand and the other from the noncoding strand. c DNA sequencing of exon 7 of the *HEXB* gene in the control sample (CC)

PCR was performed for exon 7 of *HEXB* gene for all the samples including the parent's of the dead girl and the controls (Figure 2). Sequence analysis of exon 7 of *HEXB* revealed heterozygote C → T substitution in both parent of the dead suspected girl having GM2‐gangliosidoses. Sequences of the parent (Figure 3A, B) and one of the controls have been shown in Figure 3C.

After mutation was found in exon 7 *HEXB*, other exons of *HEXB* and *HEXA* were also sequenced, but no mutation was detected in any of the other *HEXB* exons and *HEXA* exons.

**Figure 4:**
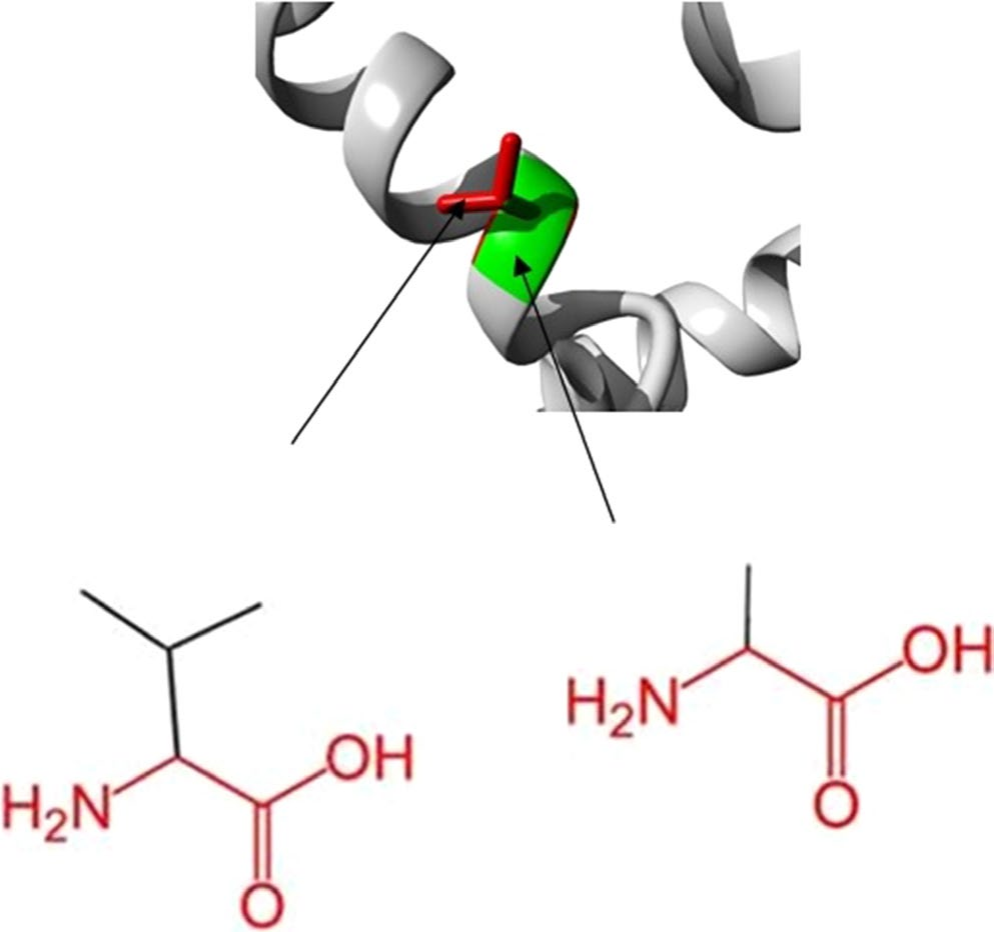
Deep view of the superimposed structure of wild‐type and mutant residue at position 278. The main protein core is shown in gray color while the wild‐type and mutated residues are shown in green and red color, respectively, and protein position 278 changed from Alanine to Valine. http://www.cmbi.ru.nl/hope/

C → T substitution resulted in amino acid change at 278 position leading to Ala to Val substitution. This alteration was detected to be deleterious and damaging with in silico tools (SIFT, PROVEAN, Polyphen‐2, I‐Mutant3.0, PHD‐SNP) )Table 4). Project Hope (http://www.cmbi.ru.nl/hope/) revealed that the wild‐type and mutant amino acids differ in their size. The wild‐type residue was buried in the core of the protein. The mutant residue is bigger and probably will not fit (Figure 4).

**Table 4:** Outputs of in silico analysis

Functional consequence	SIFT	Score	PROVEAN	Score	PolyPhen‐2	Score	I‐Mutant3.0	Stability	PhD SNP
A278V	Damaging	0.001	Deleterious	−3.74	Probably Damaging	1.000	Large decrease RI:5	−0.25	Disease

**Figure 5:**
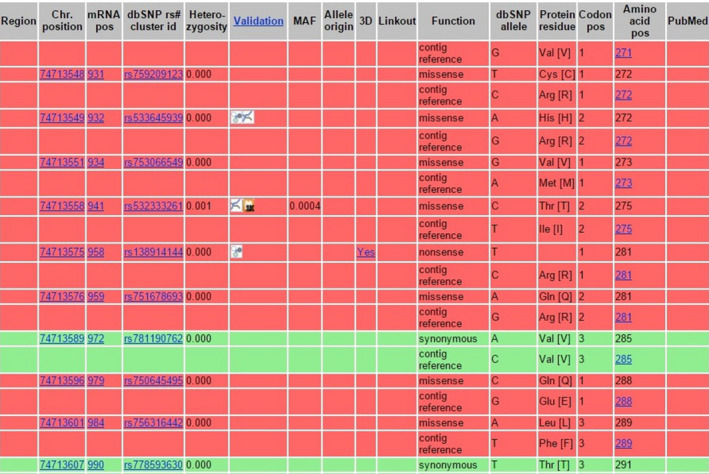
Reported SNPs flanked the position of the Ala 278 *HEXB* in the NCBI database

**Figure 6:**
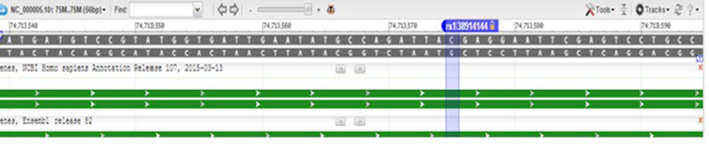
Nucleotide position 74713567 and its flanking SNPs

Inspecting the position of the mutation in the NCBI database (https://www.ncbi.nlm.nih.gov/) revealed that c.833 C>T was not a reported single nucleotide polymorphism. The results of this survey are shown in (Figure 5). Furthermore, the position of c.833 C>T mutation corresponded to the nucleotide number 74713567 and its flanking SNPs is shown in Figure 6.

## 4 DISCUSSION

In this study, we reported a novel variant in an Iranian family with a history of a dead girl with Sandhoff disease which was not found in 100 controls.

The c.833C>T is a novel mutation in exon 7 of *HEXB* that causes a substitution of Alanine 278 with Valine. Both parents were heterozygous (CT) for this mutation, and all of the control samples were homozygous (CC) in this position.

SIFT, PROVEAN, PolyPhen, and PhD‐SNP predicted that this mutation is causing disease and classified this mutation in a dangerous group of mutations. The I‐mutant analysis showed that this mutation is reducing the stability of the protein. We used 5 in silico servers because the accuracy of the in silico techniques for determining the effect (s) of mutation can be increased by combining different computational methods.^27^

HOPE simulates the structural variation between mutant and wild‐type residues. The wild‐type residue is located in a region annotated in UniProt to form an α‐helix. The mutation converts the wild‐type residue to a residue that does not prefer α‐helixes as a secondary structure (Figure 4).

Although Sandhoff disease is rare in the general population, there are several geographic regions with a high incidence of the disease including an inbred community of Metls (North American Indians), Lebanon, Creole population of Argentina. Studies have revealed that mutations occur all along this gene.^5^ Very few studies have been done in Iran^28^ on this group of diseases, and common mutations of the disease in the Iranian population are yet to be determined. Identification of common mutations in Iranian families appears essential especially for families who have a history of the disease, for genetic counseling, prenatal diagnosis, and prevention of neonatal with the disease.

*HEXB* gene is located on 5q13 and includes 14 exons.^1^ Up to now, for *HEXB* gene 88 mutations in the human mutation database have been registered.^29^ Several mutations have been detected in *HEXB* so far which causes an infantile form of the disorder; the most common mutation among them is a 16 kb deletion at the 5' end of the gene that no mRNA or β‐protein produce.^4^ Some mutations have been found in the exon 7 of *HEXB* including a nonsense mutation, c.850C˃T, which generated a stop codon at codon 284^30^ in which all of the cell lines originated from the Kennedy Institute, Baltimore, MD; a 4‐bp deletion in exon 7 (delCTTT) causes early stop codon at 273^31^ in Argentinean patients with Sandhoff disease and a single base‐pair deletion in exon 7 that change the codon for Gly‐258, GGA, and to GA^28^ in the patient with Caucasian/Indian ancestry. Finally, A missense mutation, c.821T>A, which causes p.Val274Glu^33^ has been reported in an Iranian child with juvenile Sandhoff disease.

## 5 CONCLUSION

We concluded that c.833 C>T mutation carries out almost all criteria for pathogenic mutation such as changing amino acid at the conserved location and not found in nonpatient controls. Functional studies need to confirm the results.

## CONFLICT OF INTEREST

The authors declare that they have no competing interests.

## AUTHOR CONTRIBUTIONS

FMM: participated in the design and coordination of the research study, performed experiments, and analyzed the data; FA: participated in the design and coordination of the research study, performed experiments, analyzed the data, and wrote the initial manuscript; PN: participated in the design and coordination of the research study and revised the manuscript for submission; MG: participated in the design and coordination of the research study, provided some samples, assisted in the clinical analysis, performed some experiments, and analyzed the related data; MEB: participated in the design and coordination of the research study, supervised the study, wrote the initial manuscript, and revised the manuscript for submission; all authors have seen and confirmed the final format of the manuscript.

## ETHICS APPROVAL AND CONSENT TO PARTICIPATE

The experiments were approved by the Ethics Committee of Shahrekord University.

## CONSENT FOR PUBLICATION

Written informed consent was obtained from the patient parents' for publication of their individual details and accompanying images in this manuscript. The consent form is held by the authors.

## References

[1] Sandhoff K , Conzelmann E , Neufeld E , Kaback M , Suzuki K . The GM2‐gangliosidoses In: Scriver CR, Beaudet AL, Sly WS, Valle D(eds), The metabolic basis of inherited disease. Vol 2. New York, NY: McGraw‐Hill; 1989:1807‐1839.

[2] Gravel RA , Kaback MM , Proia RL , Sandhoff K , Suzuki K , Suzuki K . The GM2‐gangliosidoses. Metab Mol Bases Inherit Dis. 2001;3:3827‐3876.

[3] Bley AE , Giannikopoulos OA , Hayden D , Kubilus K , Tifft CJ , Eichler FS . Natural history of infantile GM2 gangliosidosis. Pediatrics. 2011;128(5):e1233‐e1241. 22025593 10.1542/peds.2011-0078 PMC3208966

[4] Neote K , McInnes B , Mahuran D , Gravel R . Structure and distribution of an Alu‐type deletion mutation in Sandhoff disease. J Clin Invest. 1990;86(5):1524. 2147027 10.1172/JCI114871 PMC296899

[5] Mahuran DJ . Biochemical consequences of mutations causing the GM2‐gangliosidoses. Biochim Biophy Acta. 1999;1455(2):105‐138. 10.1016/s0925-4439(99)00074-5 10571007

[6] Chern C , Kennett R , Engel E , Mellman W , Croce CM . Assignment of the structural genes for the α subunit of hexosaminidase A, mannosephosphate isomerase, and pyruvate kinase to the region q22‐qter of human chromosome 15. Somat Cell Genet. 1977;3(6):553‐560. 341373 10.1007/BF01539065

[7] Kytzia H‐J , Sandhoff K . Evidence for two different active sites on human beta‐hexosaminidase A. Interaction of GM2 activator protein with beta‐hexosaminidase A. J Biol Chem. 1985;260(12):7568‐7572. 3158659

[8] Yasui N , Takaoka Y , Nishio H , et al. Molecular pathology of Sandhoff disease with p. Arg505Gln in HEXB: application of simulation analysis. J Hum Genet. 2013;58(9):611‐617. 23759947 10.1038/jhg.2013.68

[9] Boedecker H , Mellman W , Tedesco T , Croce CM . Assignment of the human gene for hexosaminidase B to chromosome 5. Exp Cell Res. 1975;93(2):468‐472. 1171773 10.1016/0014-4827(75)90473-5

[10] Neufeld E , Paulson J , Weinstein J , et al. Natural history and inherited disorders of a lysosomal enzyme, betahexosaminidase. J Biol Chem. 1989;264. 2525553

[11] O'Dowd BF , Klavins M , Willard H , Gravel R , Lowden J , Mahuran D . Molecular heterogeneity in the infantile and juvenile forms of Sandhoff disease (O‐variant GM2 gangliosidosis). J Biol Chem. 1986;261(27):12680‐12685. 3017984

[12] Yun Y‐M , Lee S‐N . A case refort of Sandhoff disease. Korean J Ophthalmol. 2005;19(1):68‐72. 15929490 10.3341/kjo.2005.19.1.68

[13] Der Kaloustian VM , Khoury MJ , Hallal R , et al. Sandhoff disease: a prevalent form of infantile GM2 gangliosidosis in Lebanon. Am J Hum Genet. 1981;33(1):85. 7468596 PMC1684873

[14] O'Brien JS , Okada S , Chen A , Fillerup DL . Tay‐Sachs disease: detection of heterozygotes and homozygotes by serum hexosaminidase assay. N Engl J Med. 1970;283(1):15‐20. 4986776 10.1056/NEJM197007022830104

[15] Sachs B . A Family Form of Idiocy: Generally Fatal, and Associated with Early Blindness (amaurotic Family Idiocy). 1896.

[16] Ng PC , Henikoff S . SIFT: predicting amino acid changes that affect protein function. Nucleic Acids Res. 2003;31(13):3812‐3814. 12824425 10.1093/nar/gkg509 PMC168916

[17] Choi Y , Sims GE , Murphy S , Miller JR , Chan AP . Predicting the functional effect of amino acid substitutions and indels. PLoS One. 2012;7(10):e46688. 23056405 10.1371/journal.pone.0046688 PMC3466303

[18] Ramensky V , Bork P , Sunyaev S . Human non‐synonymous SNPs: server and survey. Nucleic Acids Res. 2002;30(17):3894‐3900. 12202775 10.1093/nar/gkf493 PMC137415

[19] Capriotti E , Fariselli P , Rossi I , Casadio R . A three‐state prediction of single point mutations on protein stability changes. BMC Bioinformatics. 2008;9(2):S6. 10.1186/1471-2105-9-S2-S6 PMC2323669 18387208

[20] Capriotti E , Calabrese R , Casadio R . Predicting the insurgence of human genetic diseases associated to single point protein mutations with support vector machines and evolutionary information. Bioinformatics. 2006;22(22):2729‐2734. 16895930 10.1093/bioinformatics/btl423

[21] Adzhubei IA , Schmidt S , Peshkin L , Ramensky VE , Gerasimova A , Bork P , Kondrashov A , Sunyaev S . A method and server for predicting damaging missense mutations. Nat Methods. 2010;7(4):248–249. 10.1038/nmeth0410-248 20354512 PMC2855889

[22] Manickam M , Ravanan P , Singh P , Talwar P . In silico identification of genetic variants in glucocerebrosidase (GBA) gene involved in Gaucher's disease using multiple software tools. Front Genet. 2014;5:148 10.3389/fgene.2014.00148. Published 2014 May 27. 24904648 PMC4034330

[23] Doss CG , Magesh R . Computational identification of pathogenic associated nsSNPs and its structural impact in UROD gene: a molecular dynamics approach. Cell Biochem Biophys. 2014;70(2):735–746. 10.1007/s12013-014-9975-7 24777812

[24] Venselaar H , Te Beek TA , Kuipers RK , Hekkelman ML , Vriend G. .Protein structure analysis of mutations causing inheritable diseases. An e‐Science approach with life scientist friendly interfaces. BMC Bioinformatics. 2010;11:548 10.1186/1471-2105-11-548. Published 2010 Nov 8. 21059217 PMC2992548

[25] Mark BL , Mahuran DJ , Cherney MM , Zhao D , Knapp S , James MN . Crystal structure of human β‐hexosaminidase B: understanding the molecular basis of Sandhoff and Tay‐Sachs disease. J Mol Biol. 2003;327(5):1093‐1109. 12662933 10.1016/s0022-2836(03)00216-x PMC2910754

[26] Sakuraba H , Matsuzawa F , Aikawa S‐I , et al. Molecular and structural studies of the GM2 gangliosidosis 0 variant. J Hum Genet. 2002;47(4):176‐183. 12166653 10.1007/s100380200020

[27] Akhoundi F , Nikpour P , Emadi‐Baygi M . In silico analysis of deleterious single nucleotide polymorphisms in human BUB1 mitotic checkpoint serine/threonine kinase B gene. Meta gene. 2016;9:142‐150. 27331020 10.1016/j.mgene.2016.05.002 PMC4913181

[28] Aryan H , Aryani O , Banihashemi K , Zaman T , Houshmand M . Novel mutations in Sandhoff disease: a molecular analysis among Iranian cohort of infantile patients. Iran J Public Health. 2012;41(3):112. 23113155 PMC3481711

[29] Stenson PD , Ball EV , Howells K , Phillips AD , Mort M , Cooper DN . The Human gene mutation database: providing a comprehensive central mutation database for molecular diagnostics and personalised genomics. Human genomics. 2009;4(2):69. 20038494 10.1186/1479-7364-4-2-69 PMC3525207

[30] Zhang Z‐X , Wakamatsu N , Mules EH , Thomas GH , Gravel RA . Impact of premature stop codons on mRNA levels in infantile Sandhoff disease. Hum Mol Genet. 1994;3(1):139‐145. 8162015 10.1093/hmg/3.1.139

[31] Brown CA , McInnes B , de Kremer RD , Mahuran DJ . Characterization of two HEXB gene mutations in Argentinean patients with Sandhoff disease. Biochim Biophys Acta. 1992;1180(1):91‐98. 1390948 10.1016/0925-4439(92)90031-h

[32] McInnes B , Brown CA , Mahuran DJ . Two small deletion mutations of the HEXB gene are present in DNA from a patient with infantile Sandhoff disease. Biochim Biophys Acta. 1992;1138(4):315‐317. 1532910 10.1016/0925-4439(92)90009-c

[33] Ebrahimzadeh‐Vesal R , Hosseini S , Moghaddassian M , Abbaszadegan MR . Identification of novel missense HEXB gene mutation in Iranian‐child with juvenile Sandhoff disease. Meta Gene. 2017;12:83‐87.

